# The Prognostic Value of Deficient Mismatch Repair in Stage II–IVa Nasopharyngeal Carcinoma in the Era of IMRT

**DOI:** 10.1038/s41598-020-66678-3

**Published:** 2020-06-16

**Authors:** Fang-ming Chen, Yun-xiang Zhang, Xiu-feng Li, Jian-fang Gao, Hao Ma, Xiao-li Wang, Yang Li, Cheng Li, Ya-nan Zhang, Ya-ting Zhang, Hong-xing Kan, Han Li, Shi-geng Zhang, Fu-rong Hao, Ming-chen Wang

**Affiliations:** 1 Department of Radiation Oncology, Rongcheng People’s Hospital, Weihai, China; 2 Department of Pathology, Weifang People’s Hospital, Weifang, China; 3 Department of Radiation Oncology, Weifang People’s Hospital, Weifang, China; 4 Clinical School, Weifang Medical University, Weifang, China; 5 Department of Radiation Oncology, Yantai Yuhuangding Hospital, Yantai, China; 6 Department of Oncology, The Fourth People’s Hospital of Zibo City, Zibo, China; 7 Department of Radiation Oncology, Taian Tumour Prevention and Treatment Hospital, Taian, China; 8 Weifang Key Laboratory of Radiophysics and Oncological Radiobiology, Weifang, China

**Keywords:** Cancer, Medical research, Oncology

## Abstract

In the era of intensity-modulated radiotherapy (IMRT), it is important to analyse the prognostic value of deficient mismatch repair (dMMR) in nasopharyngeal carcinoma (NPC). In this study, in pretreatment biopsies of 69 patients with stage II–IVa NPC, the expression levels of MMR proteins, including MLH1, MSH2, MSH6 and PMS2, were assessed by immunohistochemistry (IHC). The median follow-up time was 37.5 months (3.1–87.4 months). 50.7% of cases (35/69) showed preserved expression of all 4 MMR proteins, which was interpreted as proficient mismatch repair (pMMR). Only 1.5% of cases (1/69) lost expression of all 4 MMR proteins, 26.1% of cases (18/69) have PMS2 loss alone and 21.7% of cases (15/69) lost expression of both PMS2 and MLH1. Thus, 49.3% of cases (34/69) lost expression of one or more MMR proteins, which was interpreted as dMMR. There was no significant difference (*P* > 0.05) in terms of sex, age, clinical stage, T category, N category or therapy regimens between the dMMR and pMMR groups. The multivariate Cox regression analysis revealed that dMMR was an independent significant prognostic factor for distant metastasis-free survival (DMFS) (dMMR vs pMMR: *P* = 0.01, HR = 0.25, 95% CI: 0.09~0.75). Therefore, NPC patients with dMMR had significantly superior DMFS compared with patients with pMMR. It can be expected that dMMR will become a new independent prognostic factor for NPC.

## Introduction

Nasopharyngeal carcinoma (NPC) is a kind of common head and neck cancers, occurring frequently in southern China and Southeast Asia. NPC has strong invasiveness and early cervical lymph node metastasis. Seventy percent of NPC patients have locally advanced nonmetastatic stage III-IVa disease at diagnosis. Radiotherapy remains the mainstay of treatment, which can cause many types of DNA and gene damage^1–3^. Mismatch repair (MMR) proteins play an important role in not only safeguarding genetic stability during replication, but also responding to and repairing cellular DNA damage^4–9^. In recent years, deficient MMR (dMMR) has become one of the highlights in tumour pathogenesis, disease screening, diagnosis, guiding drug use and judging prognosis, especially in colorectal cancer. Although two previous studies^10,11^ reported that dMMR was a rare event in NPC, it’s prognostic value was not analysed. In this study, we tried to explore the prognostic value of dMMR in NPC patients.

## Methods and Materials

### Patients

NPC patients were treated with intensity-modulated radiotherapy (IMRT) and were analysed retrospectively between January 1, 2012, and December 31, 2017, at the Department of Radiation Oncology of Weifang People’s Hospital (Weifang, China). This study was approved by the ethics review board of the Weifang People’s Hospital (No. KY2019003), and a waiver of informed consent was obtained due to the retrospective nature of the study. All experiments were performed in accordance with the relevant guidelines and regulations.

The inclusion criteria were as follows: (1) newly diagnosed and histologically confirmed NPC; (2) received radical IMRT during the course of treatment; (3) stage II–IVa according to the 8th edition of the American Joint Committee on Cancer (AJCC) staging system for NPC; and (4) no history of head and neck radiotherapy, other malignant tumours or severe illnesses. The exclusion criteria were as follows: (1) patients with adenocarcinoma or adenoid cystic carcinoma pathology; (2) radiotherapy not completed; (3) women who were pregnant or lactating; and (4) the biopsy sample was insufficient or severe artefacts were present. A total of 81 patients were reviewed, 12 patients were excluded, including 2 patients with keratinized squamous cell carcinoma, and 69 patients were included in the analysis. The median age was 53 years (10–82 years). All patients completed a pretreatment evaluation to identify the clinical stage, which included a complete medical history, a physical examination, haematology and biochemistry profiles, electronic fibre nasopharyngoscopy, a magnetic resonance imaging (MRI) scan of the neck and nasopharynx, chest radiography and abdominal sonography or a chest and abdominal computed tomography (CT) scan, and a whole-body bone scan using single-photon emission computed tomography; additionally, some patients underwent a PET/CT examination. All patients were restaged according to the 8th edition of the AJCC staging system^12^. The quality of the HE-stained sections and wax blocks was evaluated by 1 intermediate and 1 senior pathologist, and all histological pathologies were reviewed by them according to the World Health Organization (WHO) Classification of Head and Neck Tumours 4th Edition^13^, disagreement was resolved by discussion and consensus, and where required through discussion with a pathologist with content expertise.

### Treatment

All patients were treated with the IMRT technique, and 52 (75.4%) patients were treated with additional chemotherapy. Cisplatin-based two-drug regimens were administered to 20 (29.0%) patients as asynchronous combination chemoradiotherapy (ACCRT, including induced chemotherapy or adjuvant chemotherapy). Cisplatin alone was administered to 32 (46.4%) patients as concurrent chemotherapy (CCRT).

Target volumes and organs at risk were delineated according to the consensus^14^.The median dose of the gross tumour volume of the nasopharynx (GTVnx) was 70.0 Gy (69.0 Gy–73.0 Gy), and the median dose of the gross tumour volume of the positive cervical lymph node (GTVnd) was 68.0 Gy (66.0 Gy–70.0 Gy). The median dose of high-risk regions (CTV1) was 60.9 Gy (60.0 Gy–62.0 Gy), and the median dose of low-risk regions (CTV2) was 54.4 Gy (50.0 Gy–56.0 Gy).

### Follow-up

Primary lesions, enlarged cervical lymph nodes and acute hematologic responses were closely observed during the treatment period. The follow-up strategy was the same as previously described^15^. All events were measured from the date of the histological diagnosis^16^. The last follow-up date was November 21, 2018. Local recurrence included primary and regional nodal recurrence. The median follow-up time was 37.5 months (3.1–87.4 months). Three patients were lost to follow up, and the follow-up rate was 95.7%.

### Immunohistochemistry (IHC) staining and evaluation

The tumour tissue MMR status was detected by IHC. The formalin-fixed, paraffin-embedded (FFPE) tumour tissue blocks were obtained for IHC examination using primary antibodies against MLH1 (ES05 clone, MXB, Fuzhou, China), MSH2 (RED2 clone, ZSGB-BIO, Beijing, China), MSH6 (EP49 clone, ZSGB-BIO), and PMS2 (EP51 clone, ZSGB-BIO) according to the immunohistochemical standard operating procedure (SOP). The normal IHC staining patterns for MLH1, MSH2, MSH6 and PMS2 MMR proteins were nuclear. Samples with >25% of tumour cells stained were interpreted as expression preserved of the MMR protein^17^. dMMR was interpreted as expression loss for at least one MMR protein^18^.

### Statistical analysis

All data were analysed using SPSS statistical software (version 22.0; IBM, Armonk, NY). The chi-squared and Fisher’s exact probability tests were used to analyse differences between qualitative data. Survival curves were plotted according to the Kaplan-Meier method and were compared using the log-rank test. A Cox proportional hazards model was used to identify significant prognostic factors. Forward LR method was use to selected variables. Statistical significance was set at *P* < 0.05 (two-sided).

## Results

### Expression of MMR proteins

**Figure 1:**
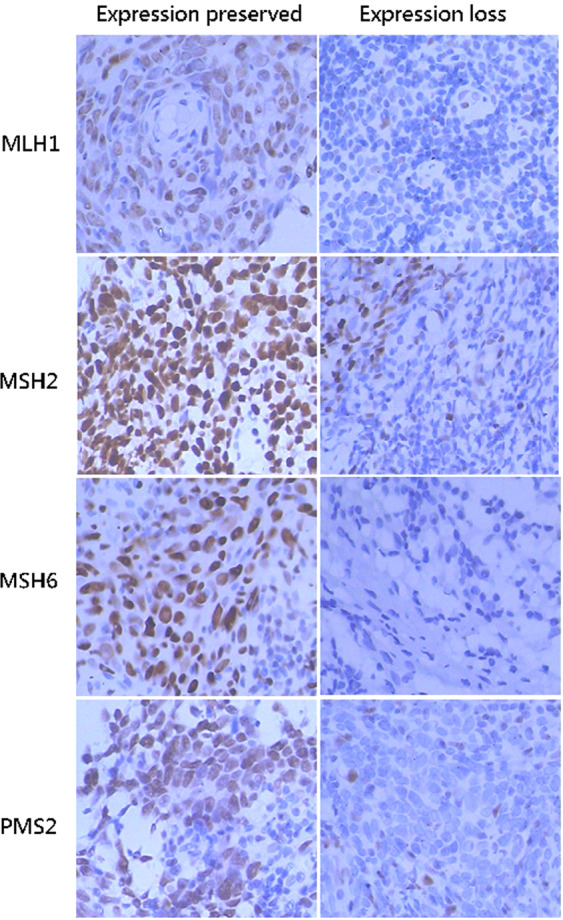
Expression of four MMR proteins in NPC tissues (×400). MMR: mismatch repair; NPC: nasopharyngeal carcinoma.

The normal IHC staining patterns for all four MMR proteins were nuclear (Fig. 1). Of 69 patients with stage II-IVa NPC, 35 (50.7%) patients showed preserved PMS2 expression, 53 (76.8%) patients showed preserved MLH1 expression, and 68 (98.6%) patients showed preserved MSH2 or MSH6 expression. For all 4 MMR proteins, 35 (50.7%) cases showed all expression preserved, which was interpreted as proficient mismatch repair (pMMR); only 1 (1.5%) cases showed loss of all expression. Eighteen (26.1%) cases were appraised as PMS2 expression loss only, while 15 (21.7%) cases showed expression loss of both PMS2 and MLH1. A total of 34 (49.3%) cases showed expression loss for at least one MMR protein, which was interpreted as dMMR (Table 1).

**Table 1:** MMR protein expression in 69 patients with NPC (n (%)).

	PMS2	MLH1	MSH2	MSH6	No. of cases
pMMR	+	+	+	+	35 (50.7)
dMMR	−	−	−	−	1 (1.5)
dMMR	−	+	+	+	18 (26.1)
dMMR	−	−	+	+	15 (21.7)
+	35 (50.7%)	53 (76.8%)	68 (98.6%)	68 (98.6%)	NA

Note: MMR: Mismatch repair; NPC: Nasopharyngeal carcinoma; dMMR: deficient MMR; pMMR: proficient MMR; + : Expression preserved; −: Expression loss; NA: Not applicable.

### Baseline characteristics and distribution of MMR

Of 69 patients with NPC, there was no significant difference (*P* > 0.05) in terms of sex, age, clinical stage, T category, N category or therapy regimens between the dMMR and pMMR groups. Histological pathology type was not analysed because only non-keratinizing squamous cell carcinoma was included (Table 2).

**Table 2:** Baseline characteristics and univariate analyses of prognostic factors of 69 patients with NPC.

Characteristic	MMR status n (%)		*χ*^2^	*P* value	OS	PFS	LRFS	DMFS
	pMMR	dMMR			*P*	*P*	*P*	*P*
Sex			1.16	0.28	0.39	0.85	0.77	0.67
Male	25 (71.4)	28 (82.4)						
Female	10 (28.6)	6 (17.6)						
Age (years)			2.44	0.12	0.07	0.02	0.04	0.04
<53	13 (37.1)	19 (55.9)						
≥53	22 (62.9)	15 (44.1)						
Histological pathology type^a^			NA	NA	NA	NA	NA	NA
Type 1	0 (0.0)	0 (0.0)						
Type 2	35 (100.0)	34 (100.0)						
Type 3	0 (0.0)	0 (0.0)						
Clinical stage^b^			0.17	1.00	0.01	0.05	0.50	0.08
II	5 (14.3)	4 (11.8)						
III	16 (45.7)	16 (47.1)						
IVa	14 (40.0)	14 (41.2)						
T-category^b^			0.01	0.95	0.02	0.03	0.08	0.04
T1-2	8 (22.9)	8 (23.5)						
T3-4	27 (77.1)	26 (76.5)						
N-category^b^			0.13	0.71	0.06	0.23	0.21	0.50
N0-2	29 (82.9)	27 (79.4)						
N3	6 (17.1)	7 (20.6)						
Therapy regimens			0.17	0.92	0.11	0.18	0.45	0.24
IMRT	8 (22.9)	9 (26.5)						
CCRT	17 (48.6)	15 (44.1)						
ACCRT	10 (28.6)	10 (29.4)						
MMR status			NA	NA	0.06	0.09	0.95	0.02
pMMR	NA	NA						
dMMR	NA	NA						

^a^WHO Classification of Head and Neck Tumours 4th Edition: type 1, Keratinizing squamous cell carcinoma; type 2, Non-keratinizing squamous cell carcinoma; type 3, Basaliod squamous cell carcinoma; ^b^According to the AJCC TNM classification of malignant tumours 8th ed.

Abbreviations: NPC: nasopharyngeal carcinoma; MMR: mismatch repair; dMMR: deficient MMR; pMMR: proficient MMR; OS: overall survival; PFS: progression-free survival; DMFS: distant metastasis-free survival; LRFS: local recurrence-free survival; IMRT: intensity-modulated radiotherapy; CCRT: concurrent chemoradiotherapy; ACCRT: asynchronous combination chemoradiotherapy; NA: Not applicable.

### Univariate analysis of prognostic factors

**Figure 2:**
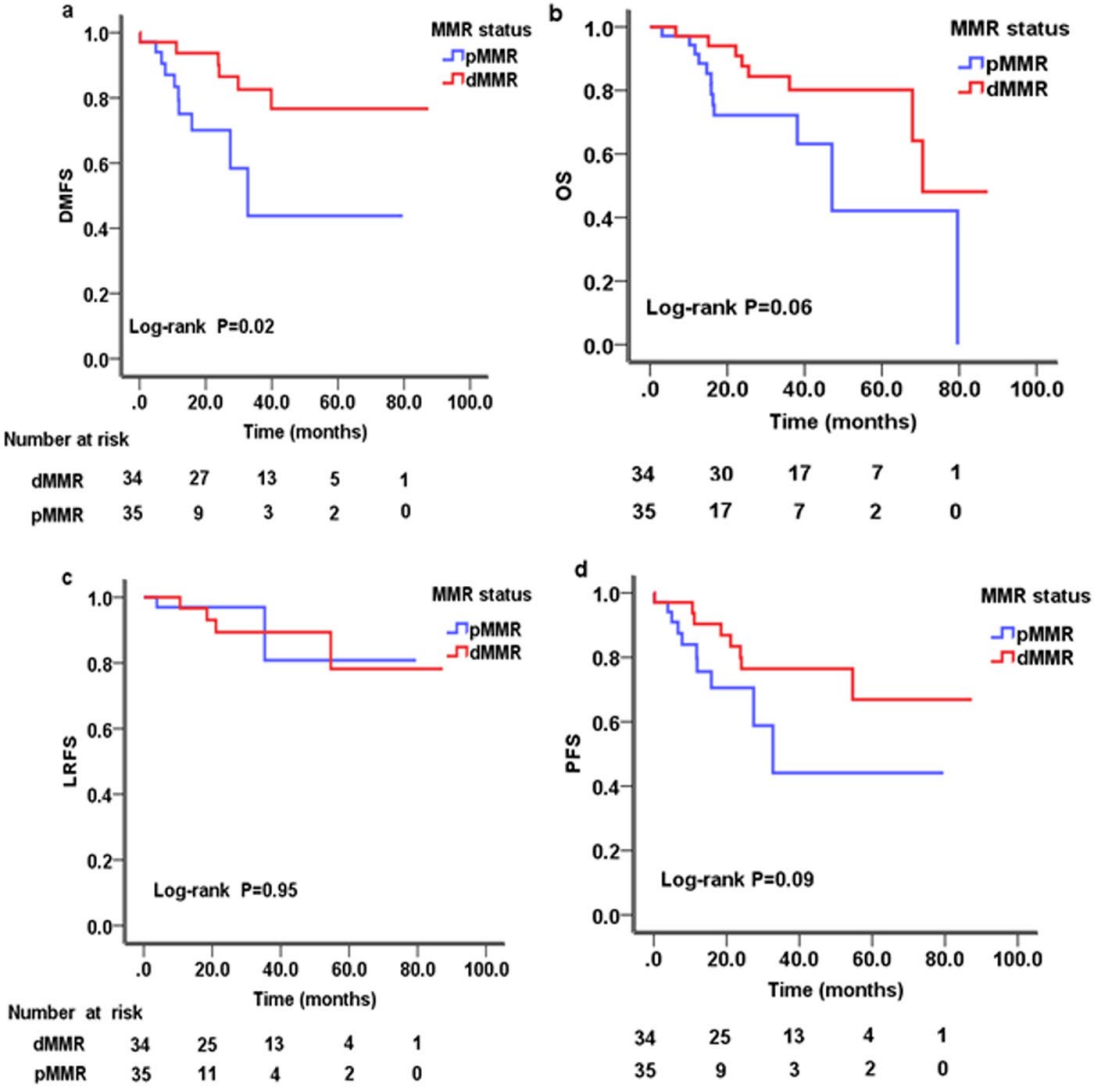
Kaplan-Meier survival curve of NPC patients according to MMR status. (**a**) Distant metastasis-free survival (DMFS); (**b**) Overall survival (OS); (**c**) Local recurrence-free survival (LRFS); (**d**) progression-free survival (PFS); NPC: nasopharyngeal carcinoma; MMR: mismatch repair; dMMR: deficient MMR; pMMR: proficient MMR.

Kaplan-Meier survival curves of NPC patients were analysed by log-rank test according to sex, age, clinical stage, T category, N-category, therapy regimens and MMR status (Table 2). The analysis revealed that clinical stage (*P* = 0.01) or T category (*P* = 0.02) for overall survival (OS), age (*P* = 0.02) or T category (*P* = 0.03) for progression-free survival (PFS), age (*P* = 0.04) for local recurrence-free survival (LRFS), and age (*P* = 0.04) or T category (*P* = 0.04) or MMR status (*P* = 0.02, Fig. 2a) for distant metastasis-free survival (DMFS) were identified as significant prognostic factors(Table 2). In addition, the influence of N category or MMR status (Fig. 2b) on OS (*P* = 0.06), and clinical stage on PFS (*P* = 0.05) were the same as the cutoff values (Table 2).

### Multivariate analysis of prognostic factors

**Figure 3:**
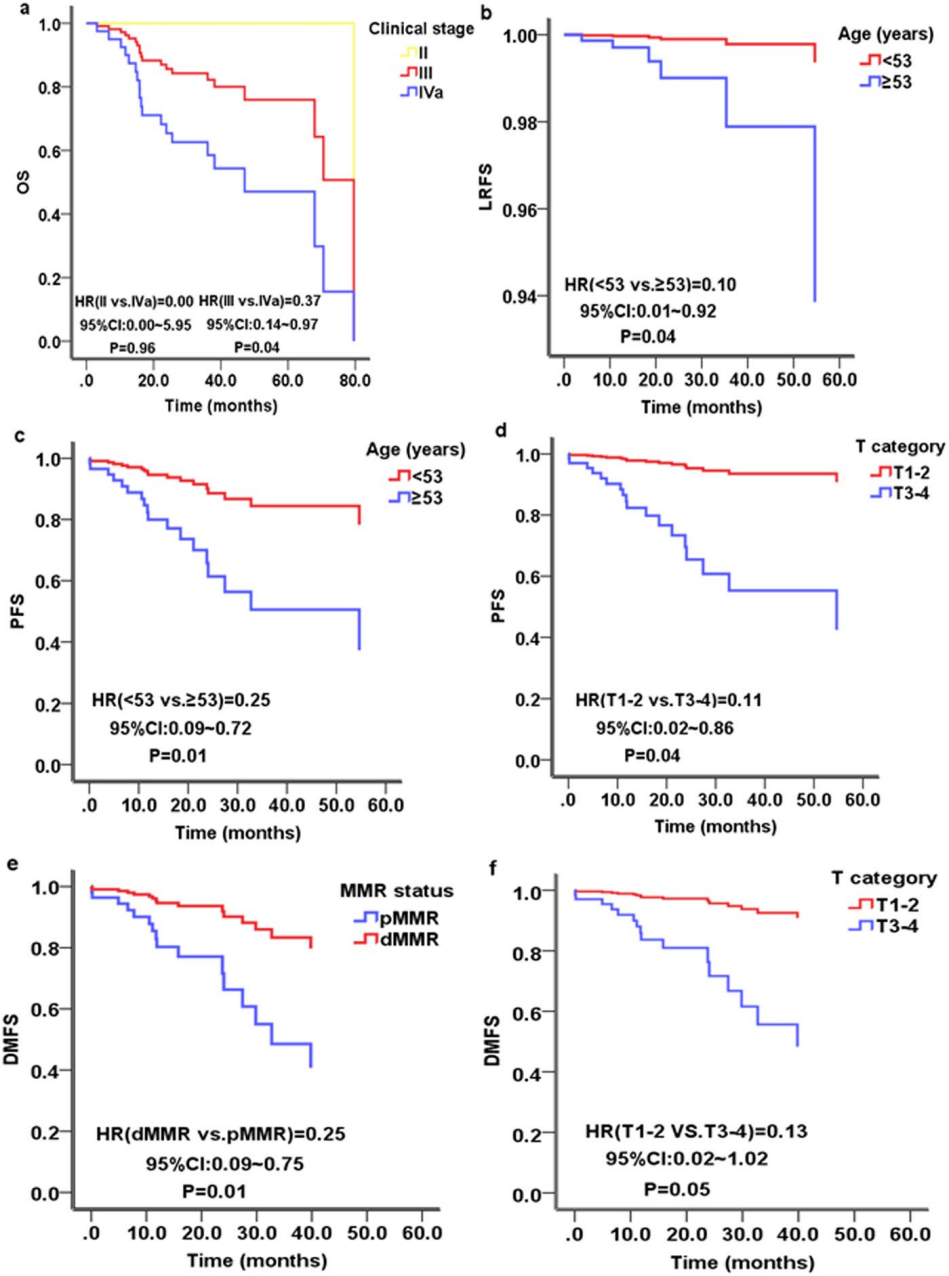
Cox proportional hazards regression model survival curve of nasopharyngeal carcinoma patients (Factors brought into the Cox proportional hazards regression model included age, clinical stage, T category, N category, MMR status and therapy strategy). (**a**) Overall survival (OS) according to clinical stage; (**b**) Local recurrence-free survival (LRFS) according to age; (**c,d**) progression-free survival (PFS) according to age or T category, respectively; (**e,f**) Distant metastasis-free survival (DMFS) according to MMR status or T category, respectively. NPC: nasopharyngeal carcinoma; MMR: mismatch repair; dMMR: deficient MMR; pMMR: proficient MMR.

The factors brought into the Cox proportional hazards regression model included age (<53 years vs. ≥53 years), clinical stage (II or III vs. IVa), T category (T1-2 vs. T3-4), N category (N0-2 vs. N3), MMR status (dMMR vs. pMMR) and therapy regimens (IMRT vs. CCRT or ACCRT). The multivariate analysis revealed that clinical stage for OS (stage III vs. IVa, *P* = 0.04, HR = 0.37, 95% CI: 0.14~0.97) (Fig. 3a, Table 3), age for LRFS (<53 vs. ≥53, *P* = 0.04, HR = 0.10, 95% CI: 0.01~0.92) (Fig. 3b, Table 3), age (<53 vs. ≥53, *P* = 0.01, HR = 0.25, 95% CI: 0.09~0.72) (Fig. 3c, Table 3) or T category (T1-2 vs. T3-4, *P* = 0.04, HR = 0.11, 95% CI: 0.02~0.86) (Fig. 3d, Table 3) for PFS, and MMR status (dMMR vs. pMMR, *P* = 0.01, HR = 0.25, 95% CI: 0.09~0.75) for DMFS (Fig. 3e, Table 3) were independent significant prognostic factors. The prognostic value of T category for DMFS was marginally statistically significant (T1-2 vs. T3-4, *P* = 0.05, HR = 0.13, 95% CI: 0.02~1.02) (Fig. 3f, Table 3).

**Table 3:** Multivariate analysis of prognostic factors in 69 patients with NPC.

Characteristic	OS			PFS			LRFS			DMFS		
	HR	95% CI	*P*	HR	95% CI	*P*	HR	95% CI	*P*	HR	95% CI	*P*
**Age (years**)*												
<53	NA	NA	NA	0.25	0.09~0.72	0.01	0.10	0.01~0.92	0.04	NA	NA	NA
≥53	Ref			Ref			Ref			Ref		
Clinical stage*^a^												
II	0.00	0.00~5.95	0.96	NA	NA	NA	NA	NA	NA	NA	NA	NA
III	0.37	0.14~0.97	0.04	NA	NA	NA	NA	NA	NA	NA	NA	NA
IVa	Ref			Ref			Ref			Ref		
T category*^a^												
T1-2	NA	NA	NA	0.11	0.02~0.86	0.04	0.00	0.00	0.98	0.13	0.02~1.02	0.05
T3-4	Ref			Ref			Ref			Ref		
N category*^a^												
N0-2	NA	NA	NA	NA	NA	NA	NA	NA	NA	NA	NA	NA
N3	Ref			Ref			Ref			Ref		
Therapy regimens*												
IMRT	Ref			Ref			Ref			Ref		
CCRT	NA	NA	NA	NA	NA	NA	NA	NA	NA	NA	NA	NA
ACCRT	NA	NA	NA	NA	NA	NA	NA	NA	NA	NA	NA	NA
MMR status*												
dMMR	NA	NA	NA	NA	NA	NA	NA	NA	NA	0.25	0.09~0.75	0.01
pMMR	Ref			Ref			Ref			Ref		

^*^Factors brought into the Cox proportional hazards regression model. ^a^According to the AJCC TNM classification of malignant tumours 8th ed.

Abbreviations: Ref: reference group; NPC: nasopharyngeal carcinoma; MMR: mismatch repair; dMMR: deficient MMR; pMMR: proficient MMR; OS: overall survival; PFS: progression-free survival; DMFS: distant metastasis-free survival; LRFS: local recurrence-free survival; IMRT: intensity-modulated radiotherapy; CCRT: concurrent chemoradiotherapy; ACCRT: asynchronous combination chemoradiotherapy; HR: hazard ratio; NA: Not applicable.

## Discussion

In living cells, MMR proteins play an important role in not only safeguarding genetic stability by excising DNA mismatches introduced by DNA polymerase during replication, but also responding to and repairing cellular DNA damage^4–8^. For a start, in human cells, MMR proteins are recruited immediately to the sites of various types of DNA damage including single-strand breaks (SSBs), double-strand breaks (DSBs) and pyrimidine dimers^4–7^, for which recruitment is mediated by protein-protein interactions in nucleotide-excision-repair dependent or function domain of MMR protein dependent manners^4^. Then, the recruitment leads to degradation of licensing factor Cdt1 (a G1-specific cell-cycle regulatory protein) in the G1 phase and efficient repair of DNA damage^6^, and apoptosis is activated via the mitochondria and p53-independent mechanisms^7^. Furthermore, MMR proteins are involved in the responses to the genotoxicity of γ-radiation in human cells, including thymidine kinase gene mutation, micronucleus formation or apoptosis, through inducing the expression of p53 and delaying the cell cycle^8^. In addition, MMR can play a role in the repair of SSBs and DSBs via homologous recombination and nonhomologous end joining^19–21^.

MMR proteins bind to mismatch errors DNA as heterodimer complexes: MSH2 binds to and cooperates with MSH6, while MLH1 binds to and cooperates with PMS2 as heterodimers. Furthermore, MSH2 and MLH1 are the dominant proteins for each pair. Hence, the loss of dominant MMR protein expression due to a pathogenic mutation or methylation is usually related to the degradation of the corresponding non-dominant partner: MSH6 is degraded if MSH2 is mutated, and PMS2 is degraded if MLH1 is mutated. However, the opposite is not true because of the compensatory effects of other MMR proteins. MSH2 expression is preserved if MSH6 is lost, and MLH1 expression is preserved if PMS2 is lost^22,23^. This law was also observed in our studies. Furthermore, in our study, the expression of MSH2 and MSH6 was loss in only one patient (1.5%), and all the MLH1/PMS2-double preserved patients (50.7%) were the same as the pMMR patients, 26.1% of the patients showed the loss of PMS2 alone. Thus, loss of PMS2 expression may be the dominant factor for the dMMR in NPC. The multivariate Cox regression analysis confirmed this hypothesis (data was not shown).

The expression of MMR proteins and the rate of dMMR were variable in different cancers or variable according to interpretation criteria^24–30^. Previous studies reported that 14.6–26.0% of patients in colorectal cancer^24,25^ and 16.0–45.0% in endometrial cancer^26–30^ were dMMR, 3.7–6.0% of patients in colorectal cancer^24,25^ and 0.0–4.2% in endometrial cancer^26–30^ showed the simultaneous loss of MSH2 and MSH6, 78.4–89.6% of patients in colorectal cancer^24,25^ and 70.2–92.6% in endometrial cancer^26–30^ showed MLH1/PMS2-double preserved, and 3.9–4.2% of patients in colorectal cancer^24,25^ and 0.5–4.9% in endometrial cancer^26–30^ showed the loss of PMS2 alone. Some studies have demonstrated that normal expression is defined as the presence of the nuclear staining of tumour cells regardless of the proportion or intensity^25–32^. However, other researchers believe that the intensity of staining and the proportion of positive cells must be considered comprehensively^17,23,24,33^. The latter approach was used in our study, and the proportion of NPC patients with dMMR was 49.3%, which was higher than the proportion in two previous reports (approximately 2%)^10,11^ on NPC. The interpretation criteria may be the major reason. Moreover, NPC is characterized by a geographical feature: the patients in the two previous studies were from southern China or the Philippines, while the patients in the current study were from northern China.

In our study, the multivariate analysis using the Cox regression model revealed that in patients with stage II-IVa NPC, clinical stage for OS (Fig. 3a, Table 3), age for LRFS (Fig. 3b, Table 3) or for PFS (Fig. 3c, Table 3), T categoryfor PFS (Fig. 3d, Table 3) were independent prognostic factors, consistent with previous reports^16,34,35^.

MMR-defective cell lines are more resistant to cell death induced by several DNA-damaging agents, including methylation agents, cisplatin and UV radiation, whereas they are more sensitive to cell death caused by interstrand crosslinking agents^4^. Radiotherapy can cause many types of DNA and gene damage, including loss of heterozygosity, homozygosity deletion^1^, DSBs^2,3^. Misrepair of radiation -induced DNA damage underlies genomic instability and increased radiosensitivity^36^; thus, MMR-defective cells may underlie increased radiosensitivity.

Previous studies have reported that compared with patients with pMMR tumours, colorectal cancer patients with dMMR tumours had significantly superior OS and PFS^24,37^, and endometrial cancer patients with dMMR tumours had significantly superior PFS^38^. In this study, there was no significant difference (*P* > 0.05) between the dMMR and pMMR groups in sex, age, clinical stage, T category, N category or therapy regimens. Univariate and multivariate analyses of the prognostic factors showed that patients with dMMR tumours had significantly superior DMFS compared with patients with pMMR tumours (dMMR vs. pMMR, *P* = 0.01, HR = 0.25, 95% CI: 0.09~0.75) (Fig. 3e, Table 3). Therefore, dMMR promises to be a potential prognostic biomarker for NPC. The better prognosis of dMMR NPC patients might result from a stronger immunologic response driven by abundant tumour infiltrating lymphocytes (TILs) in the tumour microenvironment^39^. Immunotherapy has been approved for the treatment of patients with microsatellite instability-high (MSI-H) or dMMR solid tumours in 12 different tumour types, although NPC was not included in the 12 cancers^40^. In addition, several studies confirmed that dMMR cases had reduced levels of vascular endothelial growth factor (VEGF) compared to pMMR cases, which might partly explain why patients with dMMR tumours had more favourable prognosis^39^.

In the IMRT era, distant metastasis predominates as the pattern of disease relapse in patients with stage II-IVa NPC, accounting for approximately 70% of cancer-specific mortality^41^. In this study, we found that dMMR was indicative of a favourable DMFS in patients with stage II-IVa NPC. It can be expected that dMMR will become a new independent prognostic factor of NPC. However, prospective clinical studies are needed to further investigate the prognostic value of dMMR in patients with advanced NPC and the role of dMMR in NPC immunotherapy. Thus, NPC should be screened for dMMR.

## Data Availability

The datasets generated during and/or analysed during the current study are available from the corresponding author on reasonable request.
